# *Trypanosoma cruzi* transmission dynamics in a synanthropic and domesticated host community

**DOI:** 10.1371/journal.pntd.0007902

**Published:** 2019-12-13

**Authors:** Alheli Flores-Ferrer, Etienne Waleckx, Guilhem Rascalou, Eric Dumonteil, Sébastien Gourbière

**Affiliations:** 1 UMR5096 ‘Laboratoire Génome et Développement des Plantes’, Université de Perpignan Via Domitia, Perpignan, France; 2 Institut de Recherche pour le Développement, UMR INTERTRYP IRD, CIRAD, Université de Montpellier, Montpellier, France; 3 Laboratorio de Parasitología, Centro de Investigaciones Regionales ‘Dr. Hideyo Noguchi’, Universidad Autónoma deYucatán, Mérida, Yucatán, México; 4 Department of Tropical Medicine, School of Public Health and Tropical Medicine, and Vector-Borne and Infectious Disease Research Center, Tulane University, New Orleans, Louisiana, United States of America; 5 Centre for the Study of Evolution, School of Life Sciences, University of Sussex, Brighton, United Kingdom; RTI International, UNITED STATES

## Abstract

*Trypanosoma cruzi* is the causative agent of Chagas disease, a Neglected Tropical Disease affecting 8 million people in the Americas. Triatomine hematophagous vectors feed on a high diversity of vertebrate species that can be reservoirs or dead-end hosts, such as avian species refractory to *T*. *cruzi*. To understand its transmission dynamics in synanthropic and domesticated species living within villages is essential to quantify disease risk and assess the potential of zooprophylaxis. We developed a *SI* model of *T*. *cruzi* transmission in a multi-host community where vector reproduction and parasite transmission depend on a triatomine blood-feeding rate accounting for vector host preferences and interference while feeding. The model was parameterized to describe *T*. *cruzi* transmission in villages of the Yucatan peninsula, Mexico, using the information about *Triatoma dimidiata* vectors and host populations accumulated over the past 15 years. Extensive analyses of the model showed that dogs are key reservoirs and contributors to human infection, as compared to synanthropic rodents and cats, while chickens or other domesticated avian hosts dilute *T*. *cruzi* transmission despite increasing vector abundance. In this context, reducing the number of dogs or increasing avian hosts abundance decreases incidence in humans by up to 56% and 39%, respectively, while combining such changes reduces incidence by 71%. Although such effects are only reached over >10-years periods, they represent important considerations to be included in the design of cost-effective Integrated Vector Management. The concomitant reduction in *T*. *cruzi* vector prevalence estimated by simulating these zooprophylactic interventions could indeed complement the removal of colonies from the peridomiciles or the use of insect screens that lower vector indoor abundance by ~60% and ~80%. These new findings reinforce the idea that education and community empowerment to reduce basic risk factors is a cornerstone to reach and sustain the key objective of interrupting Chagas disease intra-domiciliary transmission.

## Author summary

‘Chagas disease’ (CD) is a Neglected Tropical Disease endemic in 21 American countries that is caused by a parasite, *Trypanosoma cruzi*, transmitted to vertebrates including humans by triatomine bugs. The main strategy to restrain CD burden consists of spraying insecticides in human dwellings. However, vector control failures and emerging insecticide resistance require complementing strategies. One such strategy could be to change the human associated host community so that the abundance of parasite ‘reservoirs’ decreases and those of parasite ‘dead-ends’ increases. This requires careful quantitative evaluation using specifically tailored mathematical models. By doing so, we showed that reducing the abundance of reservoir dogs and increasing those of avian hosts that cannot be infected by *T*. *cruzi* can lower new human infections by up to 56% and 39%. Although such effects take time to manifest themselves, our results suggest that ‘zooprophylaxis’ can be part of an integrated and sustainable disease management.

## Introduction

American trypanosomiasis, also referred to as Chagas disease, is a Neglected Tropical Disease (NTD) endemic in both rural [1–3] and peri-urban/urban [4–6] areas of 21 American countries. The disease causative agent is a stercorarian protozoan parasite, *Trypanosoma cruzi*. Humans become infected mainly by contact with the faeces of bloodsucking triatomine bugs (Hemiptera, Reduviidae) infected with the parasite, although transmission through blood transfusions or from mother to child are significant components of the disease epidemiology [7]. Despite major control initiatives covering most of Latin America [8], an estimated 8 million people are currently infected with *T*. *cruzi* and another 70 million people remain at risk of infection [9]. The current international objective, set within the WHO roadmap to control, eliminate and potentially eradicate NTDs, is to interrupt intra-domiciliary transmission in the America [10]. Such an ambitious target will require innovative control strategies based on indoor residual spraying locally adjusted to the level of vector adaptation to human habitats [11] in combination with other strategies including environmental management and human habitat improvement through ecohealth approaches [12–16], zooprophylaxys [17–18], and/or drug administration [19].

As many other vector-borne human diseases, Chagas disease is a zoonosis with a strong (re-)emerging potential since *T*. *cruzi* is a generalist parasite infecting a broad range of host species and is transmitted by even more generalist triatomine vector species [20–22]. Studies have pointed out the role of host species diversity on the ecology of human vector-borne pathogens and, accordingly, on disease control [23–24]. Reservoir species can both weaken control interventions targeting the interruption of local transmission and favour the re-emergence of locally controlled parasite [25], thereby putting previous achievements into jeopardy and calling for sustained epidemiological surveillance [26–27]. On the contrary, non- or less-competent host species can dilute the transmission of generalist pathogens, which potentially makes the preservation of biodiversity a public health policy ally [28]. These two contrasting effects on pathogen transmission obviously suggest that the manipulation of local host communities could help controlling vector-borne diseases in more sustainable ways. However, the potential efficacy of such strategies is, just like vector control, likely to depend on both the ecological and socio-economical contexts [9,29–30]. The quantitative assessment of host community management then requires integrating available knowledge into eco-epidemiological models of transmission within the synanthropic and domesticated host communities that are putative targets of public health interventions.

While the empirical literature on Chagas disease ecology points at the importance of non-human hosts in the transmission of *T*. *cruzi* to human [31–33], its modelling has often been focused on vector population dynamics with the hope of optimizing vector control interventions targeting the reduction of triatomine abundance and infestation (see [34] for a review). Dynamic compartmental models of *T*. *cruzi* transmission have received less attention and they predominantly account for a single non-human host species that typically corresponds to dogs [35–38], another ‘mammalian’ or ‘rodent’ species [35,38–40], or an ‘average’ species representing the host community [41]. Avian hosts that are well-known to be refractory to *T*. *cruzi* infection [42], have been explicitly accounted for in such epidemiological models only on rare occasions [35,36,38]. Although those models provided valuable insights into the role of these typical host species in modulating the risk of infection to humans, none of them looked at their combined effects on both *T*. *cruzi* transmission and vector demography within a host community similar to what is typically identified from studies of triatomine feeding sources [43–46].

In this contribution, we studied the influence of the synanthropic and domesticated host community in the transmission of *T*. *cruzi* at a village scale using a compartmental *SI* model [47–48]. An important originality of our modelling is that vector blood-feeding rate depends on the outcome of the intraspecific competition between individuals to feed on the host community, and that this regulating process feedbacks simultaneously on vector demography and parasite transmission. The rate of vector contact has indeed been shown to be affected by indirect competitive interactions between triatomines [49–52]. These empirical studies all supported the idea that the per capita access to blood meals decreases with vector density as a result of an increase in triatomine biting perception that trigger defensive behavioural responses from their hosts. Although the consequences of such density dependent variations on vector population dynamics has been properly modelled [36], its consequences on *T*. *cruzi* transmission remain unclear. Such feedbacks are likely to be essential in assessing the effect of non-competent hosts on transmission and disease risk. The contribution of avian species to *T*. *cruzi* transmission must indeed result from a balance between their potential to increase triatomine infestation/abundance [17,53–54], by increasing blood resource and lowering vector intraspecific competition, and their ability to dilute the spread of the trypanosome by being refractory to infection [42]. We parameterized the resulting eco-epidemiological model to describe the transmission of *T*. *cruzi* by *Triatoma dimidiata* within a village of the Yucatan peninsula, Mexico. The long-term field studies carried out in this area provided key data on the local network of *T*. *cruzi* transmission that include estimates of vector and host species abundance and infection, as well as a detailed description of the triatomine blood-feeding host range. This integrative eco-epidemiological approach allowed for a systematic investigation of the effects of synanthropic and domesticated host species on the risk of transmission of *T*. *cruzi* to human. We evaluated the implications of our results for strategies of Chagas disease control that would incorporate zooprophylaxis into sustainable and efficient Integrated Vector Management.

## Materials and methods

### Modelling vector-borne transmission with vector competition and preferences for blood meals

**Fig 1:**
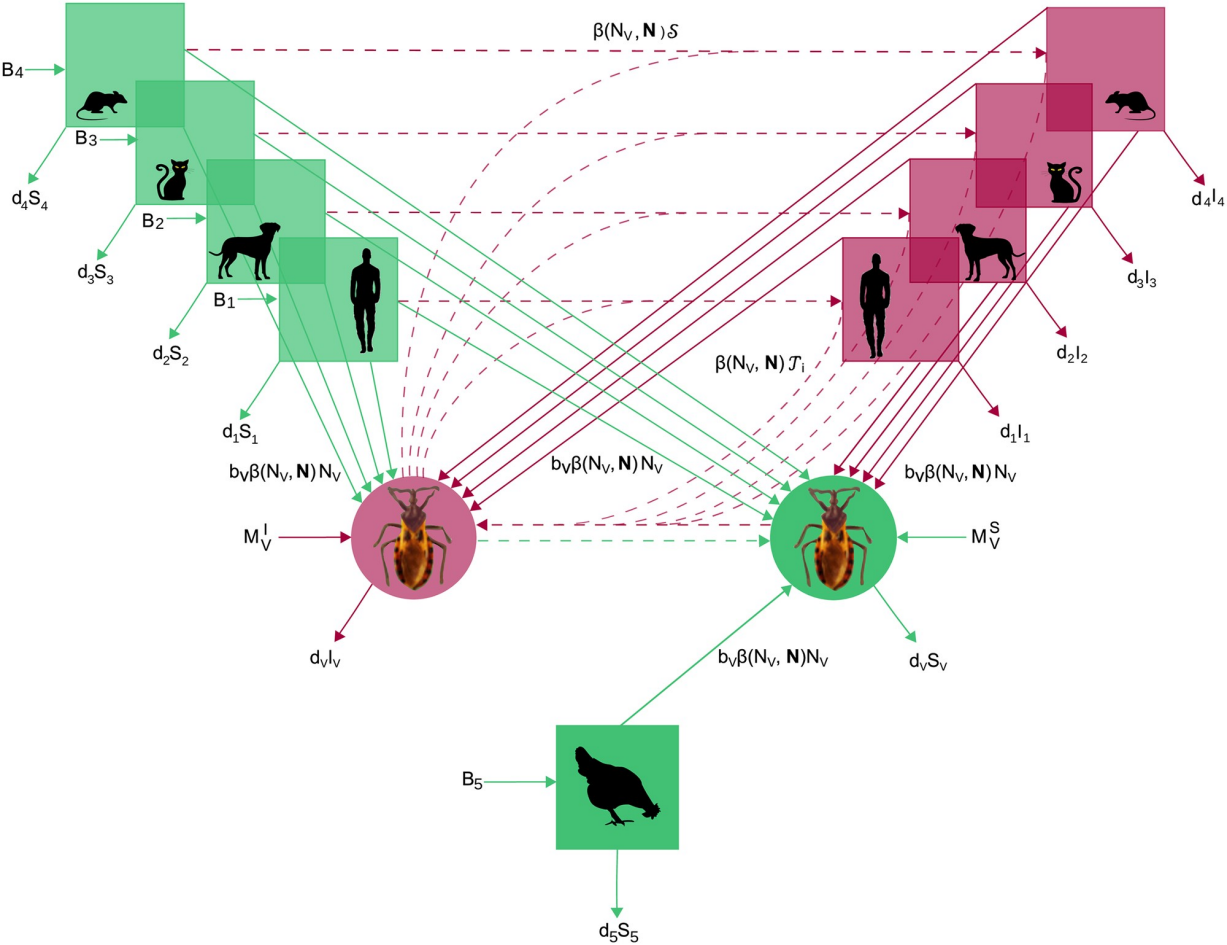
Flowchart for the SI model of *T*. *cruzi* transmission in a community of competent and non-competent hosts. Species i = 1 to 4 that are competent hosts (at the top) and vectors (in the middle) are made of susceptible (green) and infectious (red) individuals. Species 5 represents non-competent hosts (at the bottom) so that all individuals always remain susceptible (green). Arrows represent birth, death, migration and infection processes according to the parameters defined in the main text and Table 1. Continuous and dotted lines correspond to demographic and transmission processes. For simplicity, we used the notations $S=\sum_{i\in C}\phi_{i}(N)\frac{I_{i}}{N_{i}}S_{V}p_{V}$ and $T=\phi_{i}(N)\frac{S_{i}}{N_{i}}p_{iV}$.

We construct an *SI* model for the transmission of a vector-borne pathogen in a community of competent and non-competent host species (Fig 1). The novelty of this model is twofold: i) vector blood-feeding influences not only parasite transmission, but also vector reproduction, and ii) the blood-feeding rate is dependent upon intraspecific competition between vectors to access the host individuals they feed on, as suggested by field studies [49,52] and accounted for in previous triatomine population dynamics models [36]. The other processes are described according to standard vector-borne modelling. Susceptible (*S*_*i*_) and infectious (*I*_*i*_) individuals of host species *i* die at a natural per capita mortality rate *d*_*i*_ and host deaths are balanced by the recruitment of susceptible individuals at a constant rate *B*_*i*_. Susceptible (*S*_*V*_) and infectious (*I*_*V*_) vectors die at a natural per-capita mortality rate *d*_*V*_. We account for a typical constant recruitment rate that represents, in our model, vector immigration (*M*_*V*_), among which $M_{V}^{s}$ and $M_{V}^{I}$ individuals are susceptible and infected, respectively. The originality of our modelling begins with the description of the recruitment of susceptible vectors. The number of new vector individuals was considered to be the product of the fertility per blood meal *b*_*v*_ and a per vector capita blood-feeding rate *β*(*N*_*V*_, ***N***) that accounts for the competition between the *N*_*V*_ vectors aiming to feed on host individuals of all species. In this expression and thereafter, the vector **N** contains the abundance of each species of the host community. The rates of parasite transmission between hosts and vectors are concomitantly influenced by those competitive interactions as they are directly related to the per capita blood-feeding rate *β*(*N*_*V*_, ***N***). The rates of parasite transmission were also determined by the distribution of contacts between vectors and each species of the host community *ϕ*_*i*_(***N***) and the probabilities of parasite transmission per potentially infectious contact between a susceptible host of species i and an infectious vector, *p*_*iV*_, or between a susceptible vector and an infectious host, *p*_*V*_.

The model then stands as a set of ordinary differential equations describing the number of susceptible and infectious individuals that belong to the vector species;

$$\frac{{dS}_{V}}{dt}=M_{V}^{S}+b_{V}\beta (N_{V},N)N_{V}-d_{V}S_{V}-\beta (N_{V},N)\sum_{i\in C}\phi_{i}(N)\frac{I_{i}}{N_{i}}S_{V}p_{V}$$ (1)

$$\frac{{dI}_{V}}{dt}=M_{V}^{I}-d_{V}I_{V}+\beta (N_{V},N)\sum_{i\in C}\phi_{i}(N)\frac{I_{i}}{N_{i}}S_{V}p_{V}$$ (2)

and to host species *i*

$$\frac{{dS}_{i}}{dt}=B_{i}-d_{i}S_{i}-I_{V}{\beta (N_{V},N)\phi }_{i}(N)\frac{S_{i}}{N_{i}}p_{iV}\;for\;all\;i\in I$$ (3)

$$\frac{{dI}_{i}}{dt}=-d_{i}I_{i}+I_{V}{\beta (N_{V},N)\phi }_{i}(N)\frac{S_{i}}{N_{i}}p_{iV}\;for\;all\;i\in C$$ (4)

where *I* is a set of indices that allow referring to all hosts and that is partitioned into a subset of competent host species, *C* = [1,2,…,n_c_], and the complement subset of non-competent species, NC = [n_c_ +1, n_c_ +2,…,n]), with n_c_ and n denoting the total number of competent host species and the total number of host species, respectively.

The complete definition of this model requires specifying the per capita blood-feeding rate *β*(*N*_*V*_, ***N***) according to the intraspecific competition for blood meals.

The competition between vectors to feed on their hosts leads the vector per capita blood-feeding rate to be down-regulated as the ratio of vector to hosts density increases. We described this negative interaction using a standard density-dependent function [55]

$$\beta (N_{V},N)=\frac{\beta_{max}}{1+\gamma \frac{N_{V}}{N_{H}}}$$ (5)

where *β*_*max*_ stands for the maximal vector feeding rate, *γ* describes the intensity of the density-dependent regulation and *N*_*H*_ represents the ‘effective’ size of the host community. This ‘effective’ size of the community is defined as the sum of all host species abundance weighted by vector blood-feeding rates *α*_*i*_, i.e. *N*_*H*_ = Σ_i∈I_ α_i_N_i_.

### Model parameterization to *T*. *dimidiata*, *T*. *cruzi* and their domesticated and synanthropic host community in the Yucatan peninsula, Mexico

The above compartmental model was parameterized to describe the transmission of *T*. *cruzi* in rural villages of the Yucatan peninsula, Mexico, where the vector and host populations have been followed during the past 15 years. This was done in two successive steps. First, by adjusting the dimension of our model to the local network of transmission and by providing independent estimates of all its parameters, except for the probabilities of transmission per potentially infectious contact, i.e. *p*_*iV*_ and *p*_*V*_. Second, by providing indirect estimates of those last parameters that remain difficult to derive from experimental infections because of the stercorarian mode of transmission of *T*. *cruzi* [56]. We obtained these indirect estimates by fitting our model to the prevalence of *T*. *cruzi* infection observed in the vector and host species.

#### Tailoring the model to the transmission of *T*. *cruzi* by *T*. *dimidiata* in the Yucatan peninsula, Mexico

In this region, the pathogen is transmitted by non-domiciliated *T*. *dimidiata*^11^ that can potentially feed on a host community typically made of domesticated mammals (*Canis lupus familiaris*, *Felis spp*., *Bos taurus*, *Sus scrofa*) and avian species (*Meleagris gallopavo*, *Gallus gallus*), together with synanthropic rodents (*Mus musculus*, *Rattus spp*.) and birds (*Zenaida spp*.) [46,56]. This community was restricted to the 5 species identified as domestic and synanthropic hosts typically living inside the domicile and peridomicile habitats constituting the modelled village. Those species represented more than 75% of *T*. *dimidiata* blood meals according to studies of their digestive content (Moo-Millan et al., in preparation). The relative proportions of blood meals made on these different host species (*ϕ*_*i*_) were large on humans and dogs, i.e. 52% and 24%, while those taken on avian hosts, rodents and cats accounted for 11%, 7% and 6%, respectively. Accordingly, n_c_ = 4 pairs of equations of the forms of Eqs 3 and 4 were specified for each of the competent host species, with index i taking on values 1 to 4 for human, dogs, cats and rodents, respectively. One additional equation of the form Eq 3 was retained to describe the non-competent for *T*. *cruzi* avian hosts population that was referred to as i = 5. From our previous survey of 308 houses located in the same villages of the peninsula where *T*. *dimidiata* blood meals were identified, we estimated that the average abundance of human, dogs, cats, rodents, and avian hosts per house were 3.14, 0.93, 0.71, 5.86 and 4.57, respectively [54]. The numbers of hosts at the village scale were then obtained by multiplying those estimated densities by the average number of houses located in the three villages where those data were collected, which was found to be 594 according to the last regional census [57]. This led to the number of hosts of each species present in a typical village appearing in Table 1. The death rate of each host species i (d_i_) was calculated as the inverse of its average lifetime expectancy with values for human, dogs, avian, rodents and cats set to 70, 3, 0.5, 2 and 4 years, respectively [37]. The recruitment rate of host species i (*B*_*i*_) could then be estimated by assuming that the number of hosts of this species in the village equals its population dynamic equilibrium N_i_* = B_i_/d_i_, which was obtained by solving $\frac{{dS}_{i}}{dt}+\frac{{dI}_{i}}{dt}=0$ using Eqs 3 and 4 (S1 Appendix, Equ A1.5). Vectors transmitting *T*. *cruzi* in the village are either dispersers from the surrounding sylvatic environment or individuals born from the local population dynamics. The number of triatomines dispersing from the sylvatic environment into the village was derived from outcomes of the multi-model inference study by Barbu et al. [58]. We averaged the estimates derived while fitting each of the models according to the support received by those model as measured by their weight of Akaike (parameter K_s_ and quantity w_i_ in Table 2 of Barbu et al. [58]). From this standard model averaging [59], we estimated that the number of individuals daily dispersing into the village (*M*_*V*_) equals 394, and we calculated the number of those vectors that are susceptible ($M_{V}^{s}$) and infected ($M_{V}^{I}$) according to the rate of infection by *T*. *cruzi* estimated to be 0.178 (Moo-Millan et al., in preparation). To calculate the number of individuals born from the local population dynamics estimates of per vector capita blood-feeding rate (*β*(*N*_*V*_,***N***)) and vector fertility per blood meal (b_v_) are required. This first quantity in turn depends on three different estimates. First, the maximal vector feeding rate (β_max_) was set to its estimate in a metabarcoding study of vector feeding pattern performed on these *T*. *dimidiata* populations. This study concluded that individual vector blood-feeding frequency could be up to once every three days, which was found similar to earlier estimates from other species [46]. Second, the intensity at which vector individuals interact while feeding (*γ*) was set to 0.054 to ensure that their population size at equilibrium (calculated by solving Equ A1.6 in S1 Appendix) was the same as in previous village scale modelling of the population dynamics of those vectors [58]. Such a value implies that when the vector to host ratio is equal to 1/*γ*~18.5, the triatomine feeding rate is halved, which is similar to what was estimated in a previous attempt at modelling the reduction of blood meal frequency with the increase in population size [60]. Third, relative vector blood-feeding rates on host species *α*_*i*_ were estimated (by solving the non-homogeneous linear system of equations A2.2 described in S2 Appendix) to fit the proportions of blood meals made on the different host species (*ϕ*_*i*_) given their population sizes (N_i_), which lead to the values provided in Table 1. The vector fertility per blood meal was derived from Zeledón’s [61] broadly recognized experimental work on *T*. *dimidata* demography. The average amount of eggs produced over a two-years cohort study (446 eggs over 714 days [61, pages 65–67]) was converted into a production per blood meal according to the estimated number of meals made during that amount of time (238 meals). This average fertility was further weighted by the survival rate of eggs (68%) and the proportion of adult female in the triatomine population (5.9%) that were derived from these same experiments, so that it could be applied to the density of individuals (N_v_) that we modelled. Those calculations provided the value of b_v_ appearing in Table 1. Finally, the vector death rate was estimated from the average life expectancy of *T*. *dimidata* that was estimated to be 310 days [61, pages 65–67].

**Table 1:** Definition and estimates of the model parameters.

Symbol	Name	Units	Value	References
*Host community*				
	Number of host of type i	ind		
	*Human*, *Dog*, *Cat*, *Rodents*, *Avian*		1865, 552, 422, 3481, 2715	[54]
B_i_	Recruitment rate of host type i	ind.day^-1^		
	*Human*, *Dog*, *Cat*, *Rodents*, *Avian*		0.073, 0.503, 0.29, 4.765, 14.88	This study
d_i_	Death rate of host type i (10^−4^)	day^-1^		
	*Human*, *Dog*, *Cat*, *Rodents*, *Avian*		0.39, 9.13, 6.85, 13.70, 54.79	[37]
*p*_*iV*_	Probability of host i infection from vector (10^−5^)			
	*Human*, *Dog*, *Cat*, *Rodents*, *Avian*		0.06, 4.20, 9.10, 61.31, 0	This study
*Triatoma dimidiata*				
M_v_	Vector migration	ind.day^-1^	394	[58]
	*T*. *cruzi* infection prevalence in migrating vectors		0.178	Moo-Milan et al., in prep.
β_max_	Maximal vector feeding rate	day^-1^	0.333	[46]
*γ*	Density-dependent regulation of vector feeding		0.054	This study
*ϕ*_*i*_	Proportion of blood-meals on host type i			
	*Human*, *Dog*, *Cat*, *Rodents*, *Avian*		0.52, 0.24, 0.06, 0.07, 0.11	Moo-Milan et al., in prep.
*α*_*i*_	Vector feeding rates on host type i			
	*Human*, *Dog*, *Cat*, *Rodents*, *Avian*		1.53, 2.41, 0.72, 0.12, 0.22	This study
b_v_	Vector fertility per blood-meal (10^−2^)		7.58	[61]
d_v_	Vector death rate (10^−3^)	day^-1^	3.226	[61]
p_V_	Probability of vector infection from host		0.37	This study

#### Fitting the model to prevalence data

There is currently little knowledge about the probability of transmission of *T*. *cruzi* to its different host species [56], so that we had to get indirect estimates of the probabilities *p*_*iV*_ and *p*_*V*_ by fitting the model to the prevalence of *T*. *cruzi* infection observed in the vector and host populations that we modelled. Expressions of the estimate of *p*_*iV*_ and *p*_*V*_ could be found (Equ A3.1 and A3.2 in S3 Appendix) and, as expected, involved the prevalence of *T*. *cruzi* in its hosts. The prevalence values in the different host species were taken from previous field studies and were equal to 2.3% in humans (9/390) [3], 9.8% in rural dogs (10/102) [62], 8.6% in cats (19/220) [62] and 4.8% in rodents (8/165) [63]. The probabilities of transmission of *T*. *cruzi* (*p*_*iV*_ and *p*_*V*_) that were estimated from these rates of infection allowed to complete Table 1 and the parameterization of our model.

### Analysis of the transmission dynamics of *T*. *cruzi* in the Yucatan peninsula, Mexico

We first characterized the ‘standard’ dynamics of *T*. *cruzi* in the modelled village by using standard local stability analysis [64] of the dynamical model to determine the size of the vector and host population at equilibrium. We further used a next-generation approach [65] to find out the expression of R_0_ and identify the role of each host species in the overall *T*. *cruzi* transmission dynamics. We then performed systematic sensitivity analyses to identify the key determinants of the equilibrium levels of the vector and host population size and prevalence of infection with *T*. *cruzi*. Each parameter value was then varied within a range of -50% to +50% around the standard values shown in Table 1 and we recorded the resulting variation in the equilibrium level of the vector and host population size and prevalence of infection with *T*. *cruzi*. Finally, we used our modelling to simulate zooprophylactic interventions by varying the abundance of dogs and avian hosts and we assessed the resulting changes in vector population size and in the prevalence of infection of the vector and competent host species with *T*. *cruzi*.

## Results

### The standard dynamics of *T*. *cruzi* transmission in a village of the Yucatan peninsula

**Fig 2:**
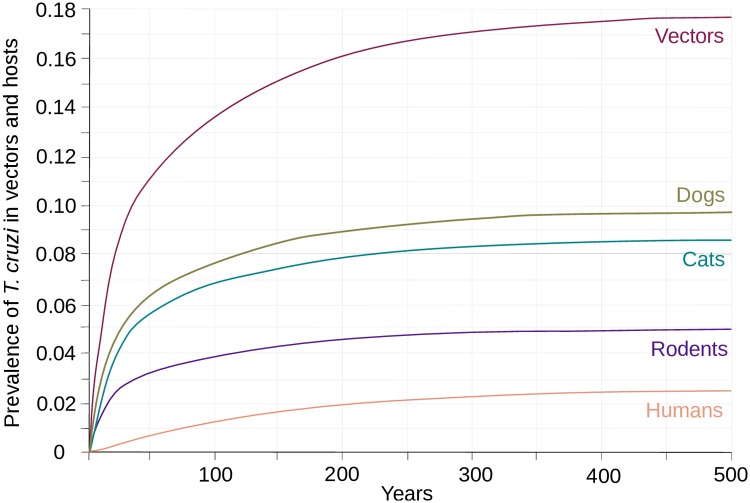
Dynamic of *T*. *cruzi* transmission in a village of the Yucatan peninsula. The dynamic of transmission was initiated by introducing parasite *T*. *cruzi* in its *T*. *dimidiata* vector. The prevalence of infection in the vector (purple) and in the competent hosts that include dogs (olive), cats (blue-green), rodents (indigo) and humans (salmon) were then followed until they reached asymptotic values corresponding to the endemic state of *T*. *cruzi* transmission typically observed in the villages of the Yucatan peninsula.

Our parameterized SI model was able to reproduce the prevalence of infection observed in the population of *T*. *dimidiata* and in its domesticated and synanthropic host community. This clearly appears in Fig 2 where *T*. *cruzi* spreads into all competent host species until it reaches the prevalence observed in the field. The estimated values of the probabilities of transmission per potentially infectious are consistent with previous estimates. After adjusting our estimate of the probability of transmission to human to account for the proportion of triatomines that are transiting in the domiciles (according to estimates provided in Barbu et al. [58]), the per contact transmission rate was indeed equal to 0.0009. This value is of the same order of magnitude as previous values estimated from triatomines found inside households [35,56,66]. Meanwhile, the relative values of the probability of transmission to human and non-human hosts is also consistent with the two previous estimates derived for opossum [67], and guinea pigs [68]. Standard stability analysis of our dynamical model allowed determining the size of the vector and host population at equilibrium (S1 Appendix). Using those theoretical results with the parameter estimates provided in Table 1 we further calculated that 84% of the vector population was born in the village, which suggests that *T*. *cruzi* could be transmitted in the village, by colonies located in the peridomiciles, even in the absence of infected vector migration from the sylvatic habitat. We then used a next-generation approach to find out the expression of R_0_ in the absence of migration (S4 Appendix). Combining this expression with our parameter estimates we obtained a R_0_ value of 1.13, which confirms that a transmission cycle of *T*. *cruzi* is sustainable in the modelled village even in the absence of vector immigration from the sylvatic habitat. Calculation of R_0_ in reduced models where only one host species was included showed that dogs, cats and rodents were reservoirs of *T*. *cruzi* that would be able to sustain local transmission as their R_0_ were equal to 1.10, 1.88 and 3.45, respectively. On the contrary, humans did not represent a sufficient host for *T*. *cruzi* as their associated R_0_ was equal to 0.65.

### Key determinants of *T*. *cruzi* transmission dynamic in its domestic and synanthropic hosts

We produced systematic sensitivity analyses around the standard parameter values presented in Table 1 to identify the key determinants of the epidemiological dynamics appearing in Fig 2. We first focused on triatomine’s demography and blood-feeding rate and preferences, before looking at the effect of the host demography and community structure.

#### Triatomine’s demography and blood-feeding rate

**Fig 3:**
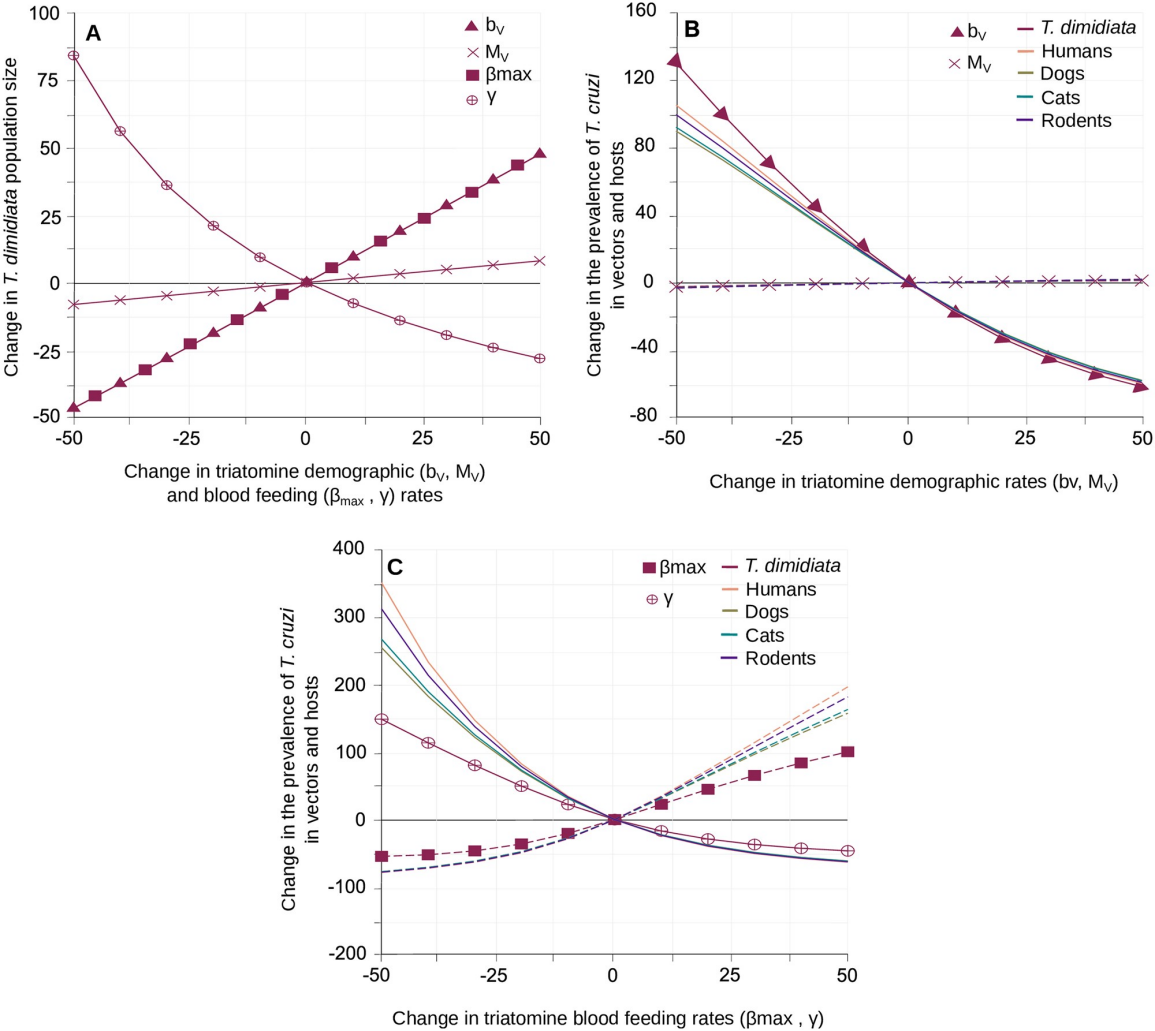
Impact of *T*. *dimidiata* demography and blood-feeding on the transmission of *T*. *cruzi*. Variation in vector population size (A) and in the prevalence of *T*. *cruzi* infection in vectors and hosts (B-C) are given with respect to changes in triatomine fertility (b_V_), immigration (M_V_), the level of competition for blood meals (*γ*) and in the maximal feeding rate (*β*_*max*_). Triangles, crosses, circles and squared stand for the effects of b_V_, M_V_, *γ* and *β*_*max*_ on *T*. *dimidiata* abundance (A) and the prevalence of infection by *T*. *cruzi* in vectors (B-C). Continuous lines describe variations in the different hosts prevalence of infection by *T*. *cruzi* according to b_V_ (B) and *γ* (C), while (superimposed) dotted lines describe variation with respect to M_V_ (B) and *β*_*max*_ (C). Host species colour code is the same as in Fig 2.

Increasing triatomine’s fertility (per blood meal, b_v_) or immigration (M_v_) lead to larger local vector populations, although the effect of the latter was substantially weaker (Fig 3A) and never allowed for the percentage of immigrant bugs found in the village to exceed 20% (S5 Appendix). Interestingly, these two demographic parameters had opposite effects on *T*. *cruzi* transmission. Higher rates of fertility were associated with a significant drop in *T*. *cruzi* prevalence in vectors and hosts, while larger arrivals of migrants steadily increased transmission, although to a much lower extent (Fig 3B). This shows the dual role of *T*. *dimidiata* immigration; migrants increase vector population size and spread parasites into the village, although those effects remain limited. By contrast, vector births significantly lower *T*. *cruzi* prevalence as newborn bugs are all considered to be susceptible. In such a context, triatomine’s fertility was a key determinant of vector population size and *T*. *cruzi* transmission by increasing the proportion of locally born susceptible vectors in the population. An essential originality of our modelling is that both parasite transmission and vector reproduction are directly determined by vector feeding rate, which itself depends on vector intraspecific competition to take blood meals upon their hosts. This explicit link between demography and blood feeding was shaped by the maximal feeding rate (*β*_*max*_) and the intensity of the competition (*γ*). The maximal feeding rate (*β*_*max*_) had the exact same positive effect on vector population size as triatomine’s fertility per blood meal (b_v_) since these two parameters multiplicatively determine the maximal number of local newborn vectors (Fig 3A). However, their impacts on parasite transmission were quite the opposite as the maximal feeding rate strengthened the prevalence of *T*. *cruzi* infection in vectors and hosts (Fig 3C) as it increased not only the vector population size, but also the frequency at which an infected (vector or host) individual made potentially infectious contacts with susceptible (host or vector) individuals. In a similar way, to increase the intensity of competition reduced the frequency of blood meals per individual, which had a negative impact on both vector population size (Fig 3A) and the prevalence of *T*. *cruzi* (Fig 3C). Noteworthy, the quantitative variations in *T*. *cruzi* prevalence produced by changes in those two parameters governing triatomine’s blood-feeding rate were consistently higher than the variations induced by those describing intrinsic demographic ability, i.e. immigration and per blood meal fertility rates. We thus looked at the effect of the parameters allowing to describe how triatomine’s blood-feeding is partitioned on the host community.

#### Vector blood-feeding preferences

**Fig 4:**
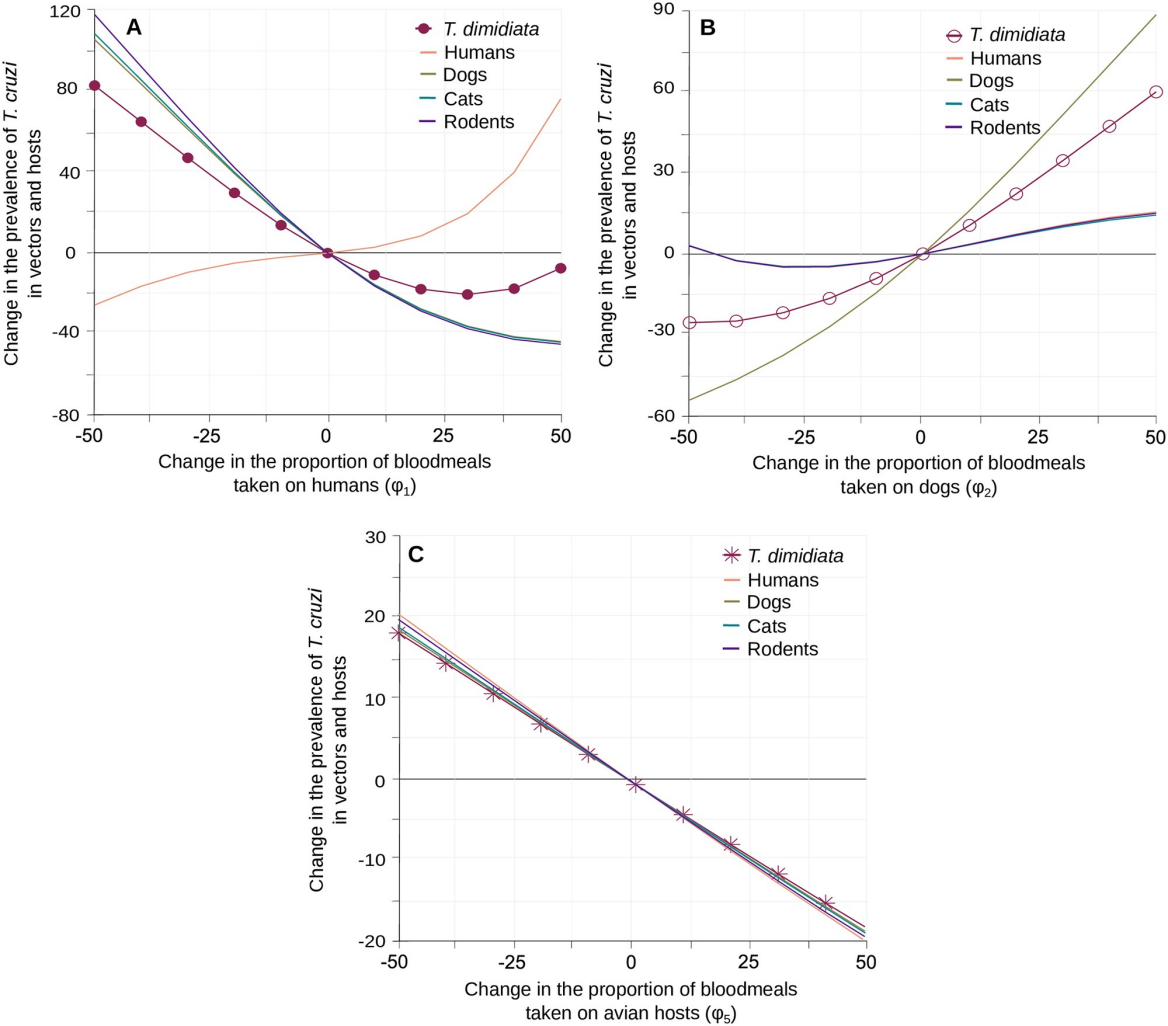
Impact of *T*. *dimidiata* blood-feeding preferences on the transmission of *T*. *cruzi*. Variation in the prevalence of *T*. *cruzi* infection in vectors and hosts are given with respect to changes in the proportion of blood meals taken on humans (A), dogs (B) and avian hosts (C). Changes in *T*. *dimidiata* prevalence of infection are indicated by circles (A-B) and crosses (C). Continuous lines describe variations in the different hosts prevalence of infection with the same host species colour code as in Fig 2.

The most important impacts of the proportion of blood meals made on the different hosts (*ϕ*_*i*_) on *T*. *cruzi* transmission were observed when modifying the vector feeding rates on human, dogs and avian hosts (Fig 4). Similar trends were observed when changing the feeding rates on cats and rodents, but the resulting changes in *T*. *cruzi* infection in vector and other competent hosts never exceeded 12% of their standard equilibrium value (S6 Appendix). When feeding frequencies on humans or dogs were increased, their *T*. *cruzi* prevalence increased steadily and substantially (Fig 4A and 4B) while vector infection showed different responses. Indeed, as the standard equilibrium value of *T*. *cruzi* prevalence in human was low (i.e. 2.3% in Table 1 and Fig 1), to increase feeding on humans initially reduced infection in vectors (Fig 4A). This effect was not apparent when increasing feeding on dogs (Fig 4B) as those are 4 times more infected than humans in the standard situation (i.e. 9.8% in Table 1 and Fig 1). In both cases, stronger increases in the proportion of blood meals made on a given host species ultimately connect that host and the vectors to such a level that the prevalence of *T*. *cruzi* infection in both species increase together in a reinforcing manner (Fig 4A and 4B). This, however, did not ensure that the prevalence of infection increased in other competent hosts. While the infectivity of dogs allowed for a strong increase in vector prevalence that spread to all other host species (Fig 4B), the rewiring of the transmission network toward human infection lead to a substantial decrease of *T*. *cruzi* prevalence in other competent hosts (Fig 4A). This difference between the contribution of human and dogs also appeared when the proportion of blood meals taken on either species was decreased. A lower rate of feeding on dogs reduced *T*. *cruzi* prevalence of infection in vectors by up to 25% (Fig 4B), while a lower rate of feeding on humans increased vector infection by 75% and doubled the prevalence of *T*. *cruzi* in all other competent hosts (Fig 4A). The pattern of variations was obviously very different when the proportion of blood meals taken on (non-competent) avian hosts increased. As expected, any increase in this proportion leads to simultaneous reductions in the prevalence of infection in the vector and all competent hosts, that can reach up to 20% (Fig 4C).

#### Host demography and community structure

**Fig 5:**
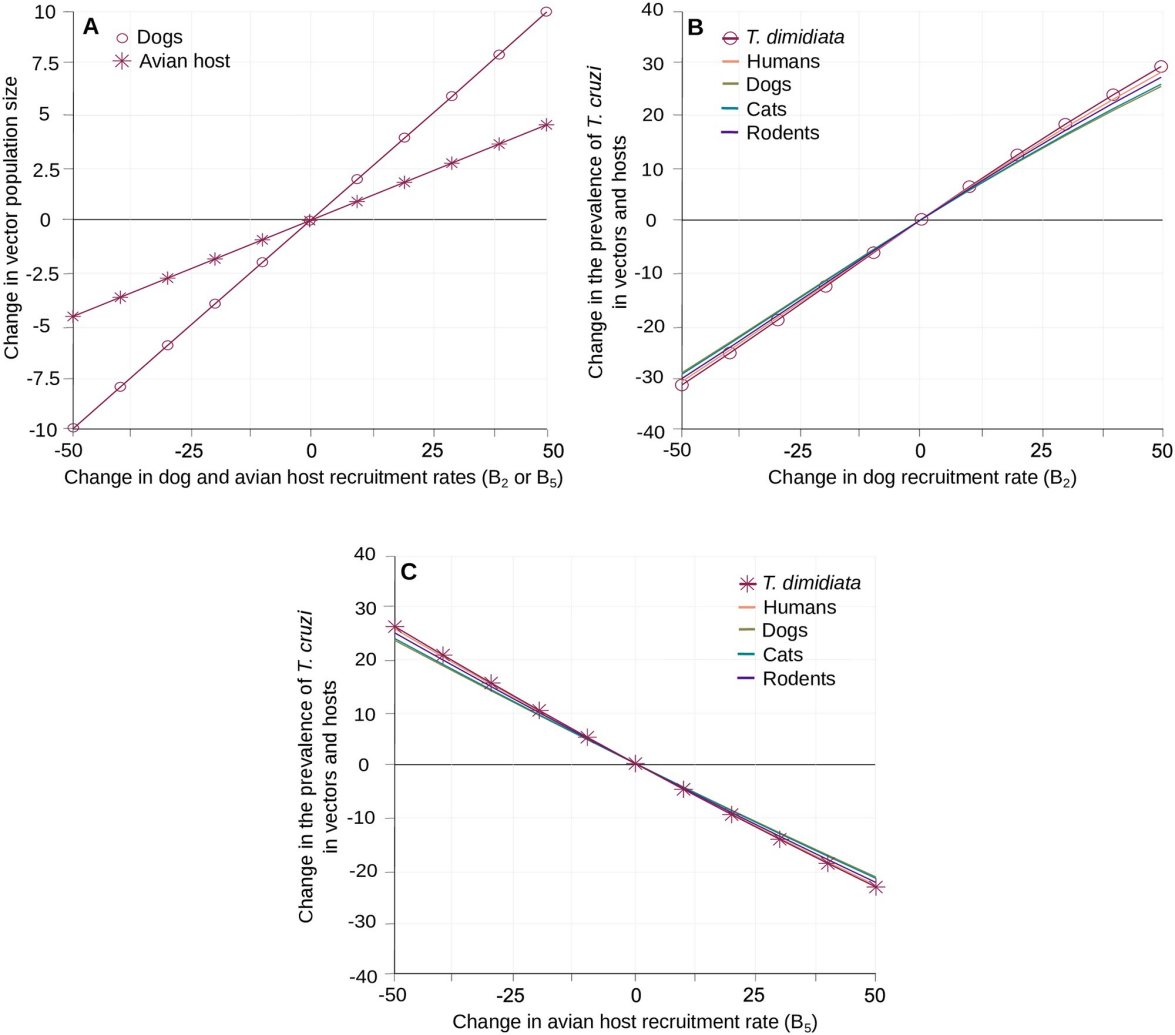
Impact of hosts demography and community structure on the transmission of *T*. *cruzi*. Variation in vector population size (A) and in the prevalence of *T*. *cruzi* infection in vectors and hosts (B-C) are given with respect to changes in the rate of recruitment (and abundance) in dogs (B_2_) and avian hosts (B_5_). Circles and crosses stand for the effects of B_2_ and B_5_ on *T*. *dimidiata* abundance (A) and its prevalence of infection by *T*. *cruzi* (B-C). Continuous lines describe variations in the different hosts prevalence of infection with the same host species colour code as in Fig 2.

The simple relationship between the host demographic rates (B_i_ and d_i_) and their abundances in the community (N_i_* = B_i_/d_i_) allowed changing the later by variations of the former. The effect of the recruitment rates (B_i_) on *T*. *cruzi* prevalence in vector and hosts are described below, while those of mortality rates (d_i_) are presented elsewhere (S7 Appendix) since changes of those mortality rates (directly linked to host life-expectancies) were considered less likely to occur in the field than changes in recruitments. Halving or doubling the standard values of B_i_ lead to directly proportional changes in the host population sizes (N_i_*). As expected, increasing the number of dogs or avian hosts in the village increased vector abundance with a maximal effect of 10% and 5%, respectively (Fig 5A). The concomitant impacts on the prevalence of *T*. *cruzi* were an increase of up to 30% and a decrease of up to 25% in host and vectors when varying dogs and avian hosts abundance, respectively (Fig 5B and 5C). Those effects were substantially larger than the 6% and 0.75% changes observed when modifying the recruitment rates of the cats and rodents populations (S7 Appendix). Noteworthy, the increase in the avian host population size resulted in a clear ‘dilution’ effect as it lowered the prevalence of *T*. *cruzi* infection in all competent hosts, despite a marked increase in the size of the triatomine population. Since the variations in *T*. *cruzi* transmission associated by changes in the size of those two typical host populations suggested that control interventions could be strengthened by changes in the composition of the domestic host community, we used our modelling to assess the potential of zooprophylactic interventions.

### Potential impacts of changes in the domestic host community on *T*. *cruzi* transmission

**Fig 6:**
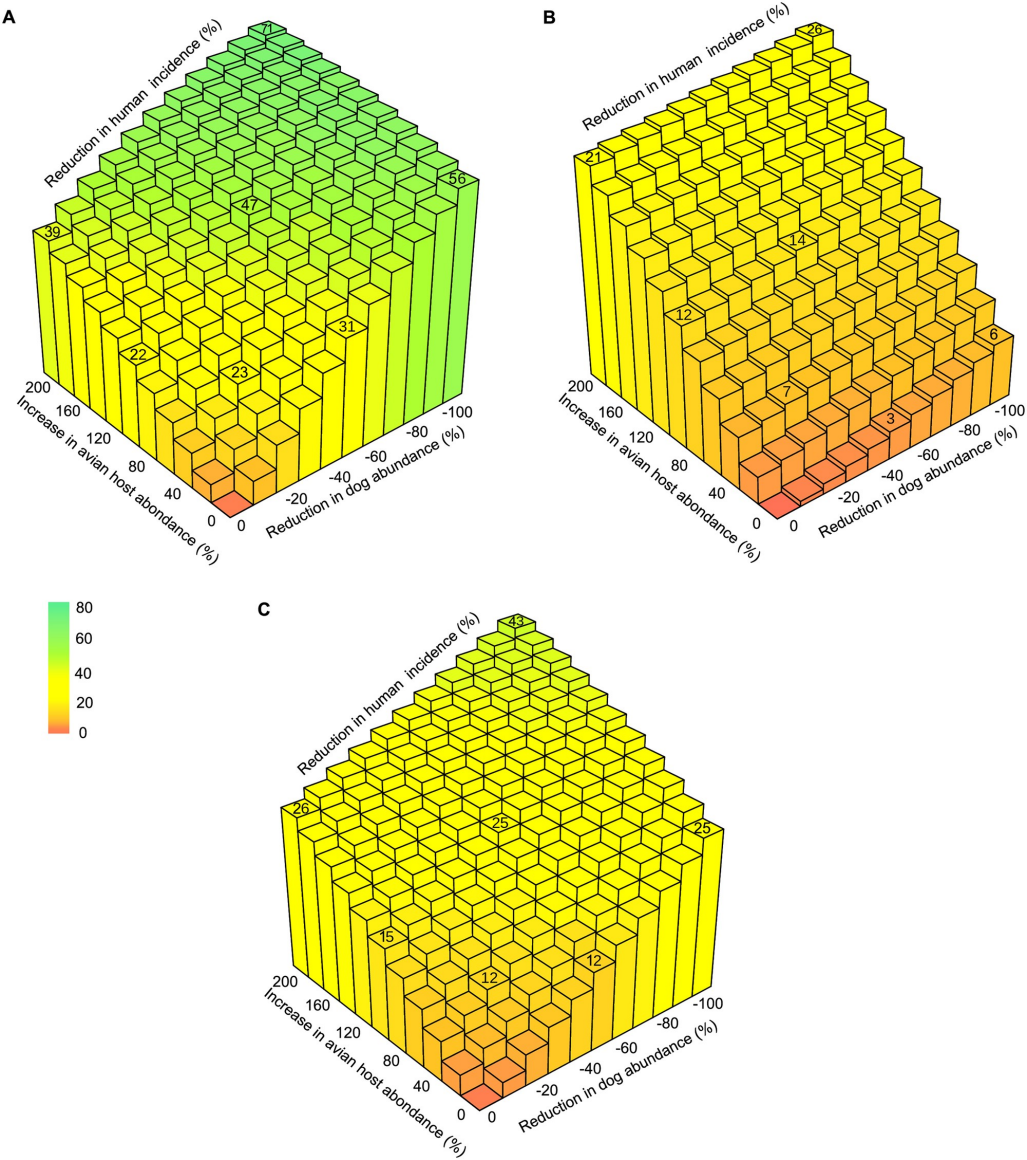
Zooprophylaxis and its potential to limit *T*. *cruzi* transmission to humans in villages of the Yucatan peninsula. The maximal potential of zooprophylactic interventions on human incidence is shown (A) along with their expected impact after 5 years (B) and 10 years (C). Interventions aim at reducing *T*. *cruzi* transmission by lowering the number of dogs (x-axis) or increasing the number of avian hosts (y-axis). The population sizes in the absence of intervention correspond to those observed in villages of the area and reported in Table 1.

We produced simulations of control interventions consisting of systematically modifying the size of the dogs and avian hosts population by changing their rate of recruitment (B_2_ and B_5_) alone or in combination. The range of variations was set so that the dog population size decreased from its observed value to 0, while the number of avian hosts was increased up to three times its standard value shown in Table 1. We measured the percentage of changes in the incidence of *T*. *cruzi* infection in human allowed by those interventions after 5 and 10 years together with their maximal (asymptotic) potential. The efficacy of removing the entire dog population or tripling the abundance of avian hosts asymptotically reached a 56% and a 39% reduction in *T*. *cruzi* incidence in human, while combining the two allowed for a reduction of up to 71% of *T*. *cruzi* transmission to humans (Fig 6A). It is important to mention that these encouraging figures can only be reached in the long term, but that a 26% and a 43% reduction in incidence can still be expected after 5 and 10-years of intervention on both dogs and avian hosts (Fig 6B and 6C). Importantly, less extreme interventions can still provide significant outcomes. Halving the size of the dog population or doubling the avian hosts population would, on their own, reduce incidence of *T*. *cruzi* infection in humans by up to 31% and 22% (Fig 6A), with expected effects after 5 and 10 years ranging in 3%-12% and 12%-15%, respectively (Fig 6B and 6C). These two changes would actually combine efficiently so that human incidence could be reduced by 47% over the long term (Fig 6A) with transitory effects of 14 and 25% after 5 and 10 years of intervention on both dogs and avian hosts (Fig 6B and 6C). Even more moderate efforts could make some difference. For instance, a 20% reduction in the number of dogs and a 50% increase in the number of avian hosts could be combined to produce a long-term reduction in the incidence of human infection by *T*. *cruzi* of 23%, with transitory effects of 7% and 12% after 5 and 10 years of control (Fig 6A–6C).

## Discussion

For decades now, studies of Chagas disease ecology have been focused on the importance of synanthropic and domesticated animals in the transmission of *T*. *cruzi* within human habitats. Various entomological risk factor analyses performed across Latin America have demonstrated that the level of house infestation by (different species of) triatomine vectors is positively associated with the presence or the abundance of dogs [17,54], cats [69–71], rodents [17,72–75], chickens [76–78] and other species of vertebrate hosts [69,73,79]. Although the contribution of those species to human infection by *T*. *cruzi* is hard to demonstrate, positive correlations have been found between the number of infected dogs and the prevalence of *T*. *cruzi* in vectors [80–81], and in human [53,82], or between seropositivity in dogs and humans [83]. Meanwhile, it has been repeatedly suggested that the presence of avian hosts, typically chickens, could reduce the prevalence of *T*. *cruzi* infection in bugs and humans [84]. While ecological and epidemiological data keep on accumulating and refining our knowledge of *T*. *cruzi* transmission networks [46,85], their integration into strategic models [86] representing *T*. *cruzi* transmission in host community remains rare. This undoubtedly limit our ability to reach key public health objectives (and to understand local failures and re-emergence) as such models are essential tools to study the potential of various control interventions aiming at interrupting *T*. *cruzi* transmission to humans according to the local specificities that can always be found in places where interventions are intended.

We developed the first *SI* model of *T*. *cruzi* transmission in a multi-host community typical of those observed in the Yucatan peninsula, Mexico, where both vector reproduction and parasite transmission depend on triatomine blood-feeding rate that itself depends on the vector’s host preferences and interference while feeding on individual hosts. By integrating all the available information about the local vector and host populations that have been accumulated over long-term field studies into this model and by producing extensive analysis of its dynamical behaviour, we provided the first evaluation of the contribution of the different hosts on the transmission of *T*. *cruzi* inside villages of the Yucatan peninsula.

The first main outcome of this integrative modelling study is that dogs are the main reservoirs of *T*. *cruzi* in the Yucatan peninsula as variations in their abundance has a 6 and 60 times higher impact on *T*. *cruzi* prevalence of infection than those of cats and rodents, respectively. Any 1% change in the number of dogs indeed resulted in a 0.6% variation in *T*. *cruzi* infection prevalence in vectors and other competent hosts, while a similar change in cats and rodents only led to 0.1% and 0.01% variations in prevalence of infection. The importance of dogs as reservoir of *T*. *cruzi* has long been recognized in other regions of Latin America (see references above) and the presence of dogs was indeed identified as a key factor of house infestation in villages of the Yucatan peninsula [54]. This study confirms that the abundance of dogs is a key determinant of *T*. *dimidiata* vector population size, with a 1% increase in the number of dogs leading to a 0.2% increase in the number of vectors. Importantly, this also demonstrates that vector abundance is a poor indicator of the risk of human infection since its variation with respect to dog abundance represented a threefold under-estimation of the variations in *T*. *cruzi* prevalence in human. The epidemiological role of cats is typically less investigated and more uncertain than the contribution of dogs [26], and the presence of cats was indeed not identified as a key factor of house infestation in the Yucatan peninsula [54]. The results of our epidemiological model support the view that the number of cats has little effect on the vector population size. However, again, this only represented an underestimation of its impact on *T*. *cruzi* infection prevalence in human that was found 2.5 higher than the effect on the abundance of local *T*. *dimidata* population, and much more significant than the effect of changes in rodent population size.

The second key outcome of our modelling is to provide evidence that avian hosts dilute the transmission of *T*. *cruzi* in the studied system. Evaluating the conditions for an increase in non-competent host abundance to dampen the transmission of infectious agents requires not only modelling the consequences of transmission failure on such hosts, but also the positive (non-linear) effects of such an increase on vector population dynamics [87]. Such demographic feedback is missing in several influential models promoting the existence of a dilution in Lyme disease [88] and is also lacking from pioneering models of *T*. *cruzi* transmission that included non-competent hosts, typically chickens, to look at their effect on domiciliary transmission [18,35,89]. By accounting for such dynamical feedback, we show that the presence of avian hosts increase the size of the *T*. *dimidiata* population with a 1% increase in the number of hosts leading to a 0.1% increase in the number of vectors. This represented just half the impact of the number of dogs on vector abundance and was very consistent with field assessment of the determinants of house infestation in the villages of the Yucatan peninsula that identified the number of chickens as a key factor although with a lower effect that dog numbers [54]. Despite such effect on the *T*. *dimidiata* population, any 1% increase in the number of avian hosts in the community lead to a 0.45% decrease in the prevalence of *T*. *cruzi* in hosts. Such an effect is consistent with a previous strategic modelling attempt that found a similarly negative, although quantitatively minor, effect of the number of chickens on the prevalence of *T*. *cruzi* infection in human [35], but contrasts with the amplification effect found elsewhere [89]. This shows that the effect of non-competent hosts in amplifying or diluting the domestic and peridomestic transmission of *T*. *cruzi* transmission can substantially vary according to the relative abundance of such hosts in the community and with the life-histories and infectiousness/infectivity of the other host species, all of those determinants being orchestrated by vector feeding behaviour. While these host features have been documented in various places across Latin America, there is a critical need to better understand vector feeding behaviour and its plasticity (in response to the host community structure [90]), to ultimately link the triatomine host feeding rate and choices to domiciliary transmission of *T*. *cruzi* using fully dynamical models that integrate increasing behavioural and ecological knowledge as exemplified for West-Nile virus [91] and initiated for Chagas disease in this contribution.

Our modelling framework could indeed be adapted to other eco-epidemiological Chagas disease contexts and provide more insights into the role of host communities on *T*. *cruzi* transmission. One obvious follow-up could be to model the ecotone area surrounding the village where other domesticated host species could potentially be targets for zooprophilaxis, especially cows that have been identified as blood meal sources in studies of *T*. *dimidiata* gut’s content [46]. Meanwhile, additional information could be derived from this emerging approach of triatomine’s feeding behaviour, such as the presence of blood from multiple hosts within the gut of a single vector individual [44,46,92], which could help to fine-tune the modelled network of transmission. Those further developments would typically require field studies and data on triatomine’s feeding rates and preferences as well as vectors’ dispersal between habitats to set up even more integrated eco-epidemiological models.

The interruption of Chagas disease intra-domiciliary transmission in the Americas will likely require the development of sustainable approaches based on Integrated Vector Management (IVM) combining vector control methods according to local ecological, biological and social conditions shaping the *T*. *cruzi* transmission network [93]. Zooprophylaxis has long been identified as a possible strategy to control Chagas disease [35], other Neglected Tropical Diseases such as cutaneous [94] and visceral leishmaniasis [95], Human African trypanosomiasis [30] and malaria [97]. The objective of zooprophylaxis is to use synanthropic or domestic animals to divert vectors from feeding upon humans, which should decrease the human-parasite contact and ultimately reduce the prevalence of infection in humans. The obvious drawback in providing additional feeding sources to the vectors is that it is likely to boost its population dynamics and population size, which might ultimately result in the opposite effect, i.e. increasing transmission to humans (zoopotentiation). The balance between zooprophylaxis and zoopotentiation has often been shown to be subtle so that a deep understanding of the local context of transmission always appears as an essential prerequisite to include the modification of the composition of host communities into integrated interventions [30,96–97]. The transmission of *T*. *cruzi* is very likely to follow the same trend and conclusions that we draw from our modelling tailored to describe transmission in the villages under study in the Yucatan peninsula are not to be generalized to other systems (as already suggested above). Still, we have shown that, in this specific context, removing dog can decrease incidence in humans by up to 56% while a threefold increase in the abundance of avian hosts can decrease it by up to 39%. Although such intervention would not have purely additive effects, such changes simultaneously applied to these two host populations could provide a 71% reduction in new human cases. While such figures could not be reached within 5 or 10-years periods, they represent very interesting long-term perspectives to design cost-effective IVM. The concomitant reduction in the vector prevalence of infection by *T*. *cruzi* that were estimated from our simulations of removing dogs, adding chickens or both interventions would indeed complement the cleaning of the peridomiciles (to eliminate established colonies) or the use of insect screens that have been shown to reduce *T*. *dimidiata* abundance by 52–62% and 87–96% at the household scale [98] and by 60% and 80% when applied to the entire village [12]. Although encouraging owners to have their dogs spayed or neutered would allow for such reduction in the production and density of susceptible reservoirs, the vaccination of dogs [99–102] to reduce their parasite carrying capacity and infectiousness to triatomine vectors (in addition to preventing cardiac disease progression in these hosts) or their protection with insecticide-impregnated collars [102] may be more readily adopted by communities. Our results taken together into simple calculations of the reduction of the force of infection [14,34,103] suggest that human incidence could be reduced by a up to 9–18 times by implementing zooprophylaxis in combination with the cleaning of the peridomiciles or the use of insect screens, respectively. The potential efficacy of these strategies reinforces the idea that education and community empowerment to reduce basic risk factors is a cornerstone to reach and sustain the key objective of interrupting intra-domiciliary transmission of Chagas Disease [13,93,104–105].

The contribution of mathematical modelling to provide either conceptual or system specific knowledge on the transmission of infectious diseases has been recognized for a long time and is being almost constantly reviewed [106–108]. The cost-effectiveness of this approach in integrating basic knowledge on pathogen transmission to estimate the potential of control strategies that cannot be tested in the field because of limited budget and/or ethical issues makes it a highly desirable tool for the control of Neglected Tropical Diseases [109–110]. The complexity of Chagas Disease eco-epidemiology, involving a broad biodiversity of triatomine vectors and vertebrate hosts and a high genetic diversity of *T*. *cruzi* strains, makes its dynamical modelling a necessary and exciting challenge to help ending the persistent burden that *T*. *cruzi* has put on human settlements and populations ever since they first colonized the Americas [111].

## Supporting information

S1 Appendix Equilibrium solutions of the SI model of *T*. *cruzi* transmission in its host community. (PDF)

S2 Appendix Estimation of vector feeding rates on host species i (*α*_*i*_). (PDF)

S3 Appendix Estimation of the probabilities of transmission of *T*. *cruzi* (*p*_*iV*_ and *p*_*V*_). (PDF)

S4 AppendixThe $R_{O}$ expression of the SI model of *T*. *cruzi* transmission in its host community.(PDF)

S5 Appendix Additional results on the effect of triatomine immigration and fertility on triatomine population. (TIF)

S6 Appendix Additional results on the effect of cats and rodents blood on *T*. *cruzi* transmission. (TIF)

S7 Appendix Additional results on the effect of hosts demography on *T*. *cruzi* transmission. (TIF)
