# Supplementary material for: Trypanosoma cruzi transmission dynamics in a synanthropic and domesticated host community
Source: PLoS Negl Trop Dis. 2019 Dec 13;13(12):e0007902. doi: 10.1371/journal.pntd.0007902 (PMC6934322; doi:10.1371/journal.pntd.0007902)
Supplement: S1 Appendix — (PDF) [file pntd.0007902.s001.pdf]

S1 Appendix : Equilibrium solutions of the SI model of *T. cruzi* transmission in its host community.

To ease the calculation of the equilibrium state of the modelled populations, the system of ordinary differential equations describing the transmission of *T. cruzi* in its host community in terms of susceptible (S_V, S_i) and infectious (I_V, I_i) individuals (Equ. 1 to 4 in the main text), was re-written by replacing the equations for the number of infectious individuals into equations describing the variations in the total number of individuals in each host (N_i) and vector (N_V) species.

$$\frac{dN_i}{dt} = B_i - d_i N_i \qquad \textbf{Equ A1.1}$$

$$\frac{dN_v}{dt} = M_V + b_V \beta(N, N_V) N_V - d_V N_V \qquad \textbf{Equ A1.2}$$

$$\frac{dS_i}{dt} = B_i - d_i S_i - I_V \beta(N_V, N)\phi_i(N)\left(\frac{S_i}{N_i}\right) p_{iV} \quad \text{for all } i \in I \qquad \textbf{Equ A1.3}$$

$$\frac{dS_V}{dt} = M_V^S + b_V \beta(N_V, N) N_V - d_V S_V - \beta(N_V, N) \sum_{i \in C} \phi_i(N) \frac{I_i}{N_i} p_V S_V \qquad \textbf{Equ A1.4}$$

with $M_V = M_V^S + M_V^I$

The calculation of the equilibrium state of this system of ordinary differential equations was achieved in four steps.

- First, the equilibrium value for the size of each host population (N_i^*) can be found by setting Equ A1.1 equals to 0, which led to:

$$N_i^* = \frac{B_i}{d_i} \qquad \textbf{Equ A1.5}$$

- Second, the equilibrium value for the size of the vector population can be found by setting Equ A1.2 equals to 0, which led to the second-degree polynomial:

$$aN_V^{*2} + bN_V^* + c = 0 \qquad \textbf{Equ A1.6}$$

where

$a = -d_V\gamma$, $b = \gamma M_v - d_V\alpha_S + b_V\alpha_S\beta_{max}$, $c = M_V\alpha_S$ and $\alpha_S = \sum_{i\in I}\alpha_i N_i^*$.

- Third, to keep on identifying the equilibrium state of the model of transmission, one further needs to find the equilibrium number of susceptible individuals in each competent host (S_i^*) and vector (S_v^*) species. This is the more complex step that involve four sub-steps.

i) Setting Equ A1.4 equals to 0, an expression of S_v^* can be found in terms of S_i^* :

$$S_v^* = \frac{D}{E + p_V\sum_{i\in C}\alpha_i S_i^*}$$ **Equ A1.7**

where

$$D = \frac{M_V^S(\alpha_S + \gamma N_V^*) + b_V\alpha_S\beta_{max}N_V^*}{-\beta_{max}} \text{ and } E = \frac{d_V(\alpha_S + \gamma N_V) + \beta_{max}p_V\sum_{i\in C}\alpha_i N_i^*}{-\beta_{max}}$$

ii) Similarly setting Equ A1.3 equals to 0, the equilibrium value of S_i^* can be written with respect to S_v^* ;

$$S_i^* = \frac{\tilde{B}_i}{S_v^* - A_i} \text{ for all } i \in C$$ **Equ A1.8**

where

$$A_i = N_V^* + \frac{d_i(\alpha_s + \gamma N_V^*)}{\beta_{max}\alpha_i p_{iV}} \text{ and } \tilde{B}_i = \frac{-B_i(\alpha_s + \gamma N_V^*)}{\beta_{max}\alpha_i p_{iV}}.$$

iii) Substituting Equ A1.8 in Equ A1.7 one can obtain an equation for S_V^*

$$S_V^* = \frac{D}{E + p_V\sum_{i\in I}\alpha_i\frac{\tilde{B}_i}{S_V^* - A_i}}$$

that is equivalent to

$$S_V^*\left(E + p_V\sum_{i\in C}\alpha_i\frac{\tilde{B}_i}{S_V^* - A_i}\right) - D = 0$$

Multiplying by $\prod_{i\in C}(S_V^* - A_i)$ in order to eliminate S_v^* from the denominator, we obtained

$$S_V^*\left(E\prod_{i\in C}(S_V^* - A_i) + p_V\sum_{i\in C}\alpha_i\frac{B_i}{S_V^* - A_i}\prod_{i\in C}(S_V^* - A_i)\right) - D\prod_{i\in C}(S_V^* - A_i) = 0$$

and after simplification

$$S_V^*\left(E\prod_{i\in C}(S_V^* - A_i) + p_V\sum_{i\in C}\alpha_i\,B_i\prod_{j\in C,j\neq i}\left(S_V^* - A_j\right)\right) - D\prod_{i\in C}(S_V^* - A_i) = 0$$

This ultimately allowed to obtain the polynomial equation to be solved to identify the equilibrium number of susceptible vectors (S_V^*);

$$(S_V^*E - D)\prod_{i\in C}(S_V^* - A_i) + p_V S_V^*\sum_{i\in C}\left(\alpha_i B_i\prod_{j\in C,j\neq i}(S_V^* - A_j)\right) = 0 \qquad \textbf{Equ A1.9}$$

and whose degree corresponds to the number of competent species plus one, i.e. n_c+1.

iv) To numerically solve this equation, e.g. by using the package ‘polynom’ in R (R Core Team 2018), provides the solution S_V^* that can then be used to further calculate S_i^* according to Equ A1.8.

- Finally, the number of infectious (I_V^*, I_i^*) individuals are simply obtained by calculating the difference between the total number of individuals at equilibrium (N_V^*, N_i^*) and the number of susceptible individuals (S_V^*, S_i^*) in each population.
