# Supplementary material for: Trypanosoma cruzi transmission dynamics in a synanthropic and domesticated host community
Source: PLoS Negl Trop Dis. 2019 Dec 13;13(12):e0007902. doi: 10.1371/journal.pntd.0007902 (PMC6934322; doi:10.1371/journal.pntd.0007902)
Supplement: S2 Appendix — (PDF) [file pntd.0007902.s002.pdf]

S2 Appendix : Estimation of vector feeding rates on host species i (α_i).

The proportion of blood meals that triatomines take on host species i (ϕ_i) can be linked to the relative feeding rates on host species i (α_i) and the hosts abundance in the community (N_i) using the standard relationship (Stone and Chitnis 2015):

$$\phi_i(N) = \frac{\alpha_i N_i}{\sum_{i \in I} \alpha_i N_i}, \text{ for all } i \in I \qquad \textbf{Equ A2.1}$$

The set of equations A2.1 can be re-written as follows:

$$\alpha_i N_i(\phi_i - 1) + \sum_{j=1, j \neq i}^{n} \alpha_j N_j = 0 \text{ for all } i \in I \qquad \textbf{Equ A2.2}$$

where $\sum_{i \in I} \alpha_i = n$.

The matrix representation of the corresponding non-homogeneous system of n linear equations is

$$\begin{pmatrix} N_1(\phi_1 - 1) & N_2\phi_1 & \cdots & N_n\phi_1 \\ N_1\phi_2 & N_2(\phi_2 - 1) & \cdots & N_n\phi_2 \\ \vdots & \vdots & \cdots & \vdots \\ N_1\phi_{n-1} & N_2\phi_{n-1} & \cdots & N_n\phi_{n-1} \\ 1 & 1 & \cdots & 1 \end{pmatrix} \begin{pmatrix} \alpha_1 \\ \alpha_2 \\ \vdots \\ \alpha_{n-1} \\ \alpha_n \end{pmatrix} = \begin{pmatrix} 0 \\ 0 \\ \vdots \\ 0 \\ n \end{pmatrix}$$

and it can be solved to estimate the model parameters α_i according to the field estimates of ϕ_i (the proportions of blood meals taken on each host species) and N_i (the abundance of each host species in the community). We used the basic linear algebra package implemented in the R environment (R Core Team 2018) to solve this linear system and find the estimates of α_i appearing in table 1.
