# Supplementary material for: Trypanosoma cruzi transmission dynamics in a synanthropic and domesticated host community
Source: PLoS Negl Trop Dis. 2019 Dec 13;13(12):e0007902. doi: 10.1371/journal.pntd.0007902 (PMC6934322; doi:10.1371/journal.pntd.0007902)
Supplement: S3 Appendix — (PDF) [file pntd.0007902.s003.pdf]

S3 Appendix : Estimation of the probabilities of transmission of *T. cruzi* (p_{iV} and p_V).

The probabilities of transmission can be estimated from the equilibrium level of the system of ordinary differential equations describing the transmission of *T. cruzi*.

Specifically, setting equation A1.3 equals to 0, straightforward calculations allow to find the expression of the estimate of p_{iV} :

$$p_{Vi} = \frac{B_i - d_i S_i^*}{I_V^* \beta(N_V^*, N^*) \phi_i(N^*) \frac{S_i^*}{N_i^*}} \text{ for all } i \in C. \qquad \textbf{Equ A3.1}$$

Similarly, setting equation A1.4 at equilibrium and simple calculations provide the expression of the estimate of p_V :

$$p_V = \frac{M_V^S + b_V \beta(N_V^*, N^*) N_V^* - d_V S_V^*}{\beta(N_V^*, N^*) \sum_{i \in C} \left(\phi_i(N^*) \frac{I_i^*}{N_i^*} \right) S_V^*}. \qquad \textbf{Equ A3.2}$$
