# Supplementary material for: Trypanosoma cruzi transmission dynamics in a synanthropic and domesticated host community
Source: PLoS Negl Trop Dis. 2019 Dec 13;13(12):e0007902. doi: 10.1371/journal.pntd.0007902 (PMC6934322; doi:10.1371/journal.pntd.0007902)
Supplement: S4 Appendix — (PDF) [file pntd.0007902.s004.pdf]

S4 Appendix : The $\Re_O$ expression of the SI model of *T. cruzi* transmission in its host community.

We used the 'Next Generation' approach (Diekmann et al., 2010) to identify the expression of $\Re_O$ in the SI model of transmission of *T. cruzi* in its host community that is described by Equ. 1 to 4 in the main text.

To derive the expression of $\Re_O$, we focus on the equations that in our system of ordinary differential equations describe the variations in the number of vector and host infected individuals, i.e. Equ 2 and 4 described in the main text;

$$\frac{dI_V}{dt} = \mathrm{M}_V^I - d_V I_V + \beta(N_V, \boldsymbol{N}) \sum_{i \in C} \phi_i(\boldsymbol{N}) \frac{I_i}{N_i} S_V p_V$$

$$\frac{dI_i}{dt} = -d_i I_i + I_V \beta(N_V, \boldsymbol{N}) \phi_i(\boldsymbol{N}) \frac{S_i}{N_i} p_{iV} \qquad \text{for all } i \in C$$

At the disease-free equilibrium, $N_i = S_i$, $N_v = S_v$ and further considering $M_V^I = 0$, this subsystem (also known as the linearized infection subsystem) becomes:

$$\frac{dI_V}{dt} = -d_V I_V + \beta(N_V, \boldsymbol{N}) \sum_{i \in C} \phi_i(\boldsymbol{N}) \frac{I_i}{N_i} N_V p_V$$

$$\frac{dI_i}{dt} = -d_i I_i + I_V \beta(N_V, \boldsymbol{N}) \phi_i(\boldsymbol{N}) p_{iV} \qquad \text{for all } i \in C$$

As explained in Diekmann et al. (2010), this system of ordinary differential equations can be represented by a matrix T made of transmission terms and a matrix Σ containing transition terms;

$$T = \beta(N_V, \boldsymbol{N}) \begin{pmatrix} 0 & p_V \phi_1(\boldsymbol{N}) \frac{N_V}{N_1} & \cdots\cdots & p_V \phi_{n_c}(\boldsymbol{N}) \frac{N_V}{N_{n_c}} \\ \phi_1(\boldsymbol{N}) P_{1V} & 0 & \cdots\cdots & 0 \\ \vdots & \vdots & \ddots & \vdots \\ \phi_{n_c}(\boldsymbol{N}) P_{n_c V} & 0 & \cdots\cdots & 0 \end{pmatrix}$$

and

$$\Sigma = -\begin{pmatrix} d_V & 0 & \cdots & 0 \\ 0 & d_1 & \cdots & 0 \\ \vdots & \vdots & \ddots & \vdots \\ 0 & 0 & \cdots & d_{n_c} \end{pmatrix}.$$

The expression of $\Re_O$ can then be found by identifying the spectral radius (the dominant eigenvalue) of the matrix $K_L = -T\Sigma^{-1}$ where K_L is called the next generation matrix with large domain.

Since Σ is a diagonal non-singular square matrix, its inverse is:

$$\Sigma^{-1} = -\begin{pmatrix} \frac{1}{dv} & 0 & \cdots & 0 \\ 0 & \frac{1}{d_1} & \cdots & 0 \\ \vdots & \vdots & \ddots & \vdots \\ 0 & 0 & \cdots & \frac{1}{d_{n_c}} \end{pmatrix}$$

and basic algebraic manipulations allow to show that the next generation matrix with large domain writes:

$$K_L = -T\Sigma^{-1} = \beta(N_V, \boldsymbol{N}) \begin{pmatrix} 0 & p_V\phi_1(\boldsymbol{N})\frac{N_V}{N_1} & \cdots & p_V\phi_{n_c}(\boldsymbol{N})\frac{N_V}{N_{n_c}} \\ \frac{1}{d_V}\phi_1(\boldsymbol{N})p_{1V} & 0 & \cdots & 0 \\ \vdots & \vdots & \ddots & \vdots \\ \frac{1}{d_V}\phi_{n_c}(\boldsymbol{N})p_{n_cV} & 0 & \cdots & 0 \end{pmatrix}.$$

In order to compute the eigenvalues of K_L, we need to find the solutions of $|K_L - \lambda I| = 0$.

It can be shown that every n dimensional square matrix of the form:

$$A = \begin{pmatrix} -\lambda & a_1 & & a_n \\ b_1 & -\lambda & & 0 \\ b_2 & 0 & \cdots & 0 \\ \vdots & \vdots & & \vdots \\ b_n & 0 & & -\lambda \end{pmatrix}$$

has as determinant $|A| = (-1)^n \lambda^n + (-1)^{n+1} \lambda^{n-2} \sum_{i=1}^{n} a_i \, b_i$.

Since $K_L - \lambda$ corresponds to such a square matrix of dimension $(n_C + 1)$ and because $|kA - \lambda I| = k^n \left| A - \frac{\lambda}{k} I \right|$ for any non-null real value of k, one can show that

$$|K_L - \lambda I| = \beta^{n_c+1}(N_V, \boldsymbol{N}) \left[(-1)^{n_c+1} \left(\frac{\lambda}{\beta(N_V, \boldsymbol{N})} \right)^{n_c+1} + (-1)^{n_c} \left(\frac{\lambda}{\beta(N_V, \boldsymbol{N})} \right)^{n-1} \frac{N_V}{d_V} p_V \sum_{i \in C} \left(\phi_i^2(\boldsymbol{N}) \frac{p_{iV}}{d_i N_i} \right) \right].$$

As we look at the solutions of $|K_L - \lambda I| = 0$, it comes:

$$(-1)^{n_C+1} \left(\frac{\lambda}{\beta(N_V, \boldsymbol{N})} \right)^{n_C+1} + (-1)^{n_C} \left(\frac{\lambda}{\beta(N_V, \boldsymbol{N})} \right)^{n-1} \frac{N_V}{d_V} p_V \sum_{i \in C} \left(\phi_i^2(\boldsymbol{N}) \frac{p_{iV}}{d_i N_i} \right) = 0.$$

Reducing and factoring the above equality, one find it equivalent to:

$$-\left(\frac{\lambda}{\beta(N_V, \boldsymbol{N})} \right)^{n_C-1} \left(\left(\frac{\lambda}{\beta(N_V, \boldsymbol{N})} \right)^2 - \frac{N_V}{d_V} p_V \sum_{i \in C} \left(\phi_i^2(\boldsymbol{N}) \frac{p_{iV}}{d_i N_i} \right) \right) = 0,$$

whose following non-trivial solution corresponds to the expression of $\Re_0$:

$$\Re_0 = \sqrt{\sum_{i \in C} \beta^2(N_V, \boldsymbol{N}) \phi_i^2(\boldsymbol{N}) \frac{p_{iV} p_V N_V}{d_i d_V N_i}}.$$
