# Supplementary figures and images for: Trypanosoma cruzi transmission dynamics in a synanthropic and domesticated host community
Source: PLoS Negl Trop Dis. 2019 Dec 13;13(12):e0007902. doi: 10.1371/journal.pntd.0007902 (PMC6934322; doi:10.1371/journal.pntd.0007902)

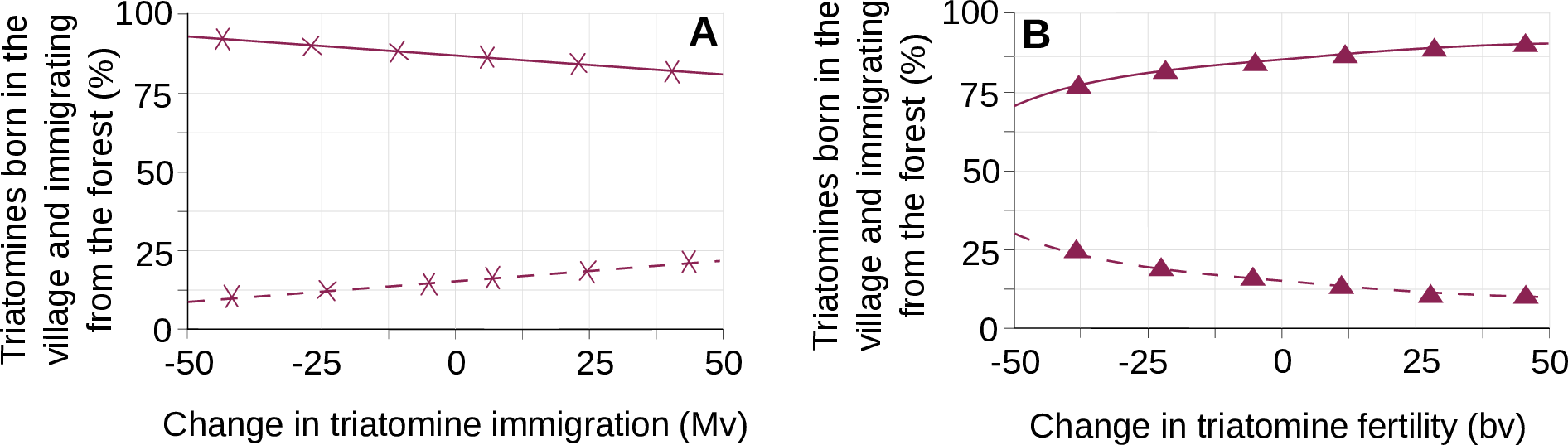

Supplement: S5 Appendix — (TIF) [file pntd.0007902.s005.tif]

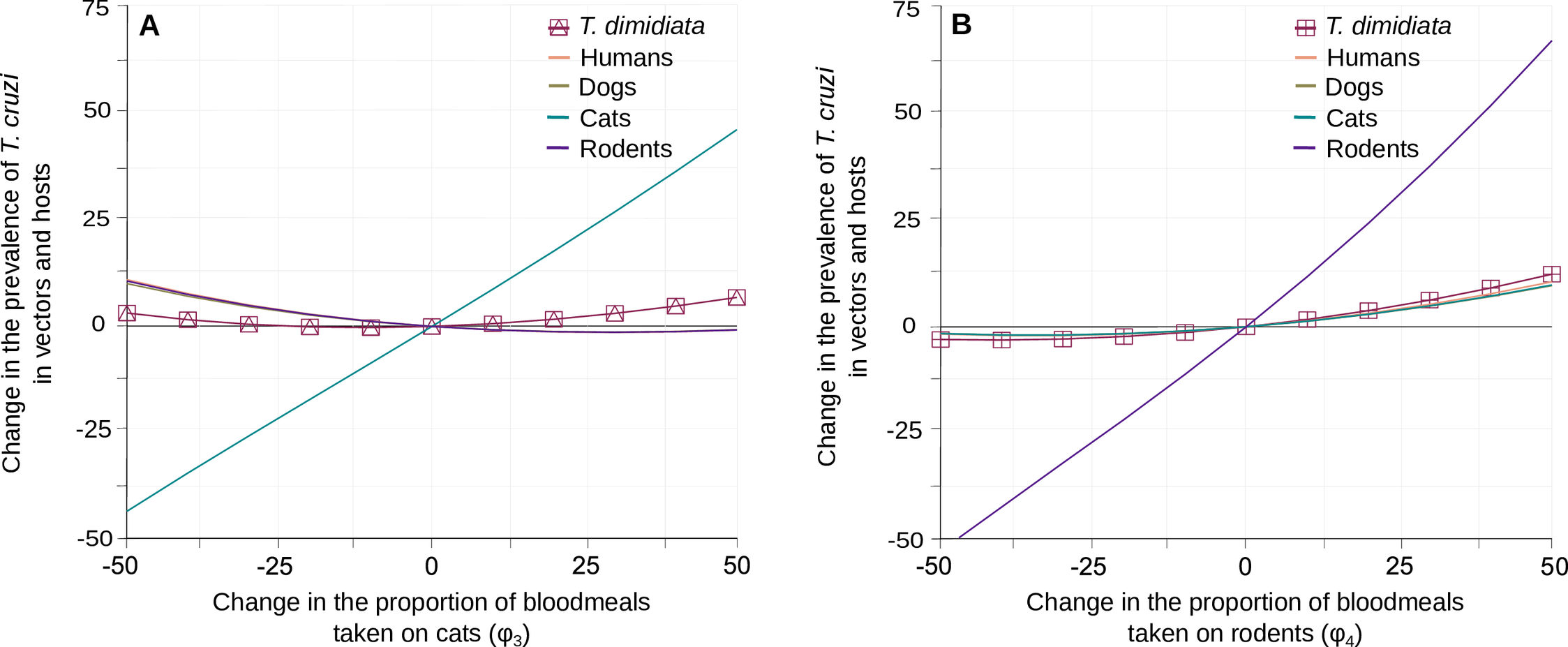

Supplement: S6 Appendix — (TIF) [file pntd.0007902.s006.tif]

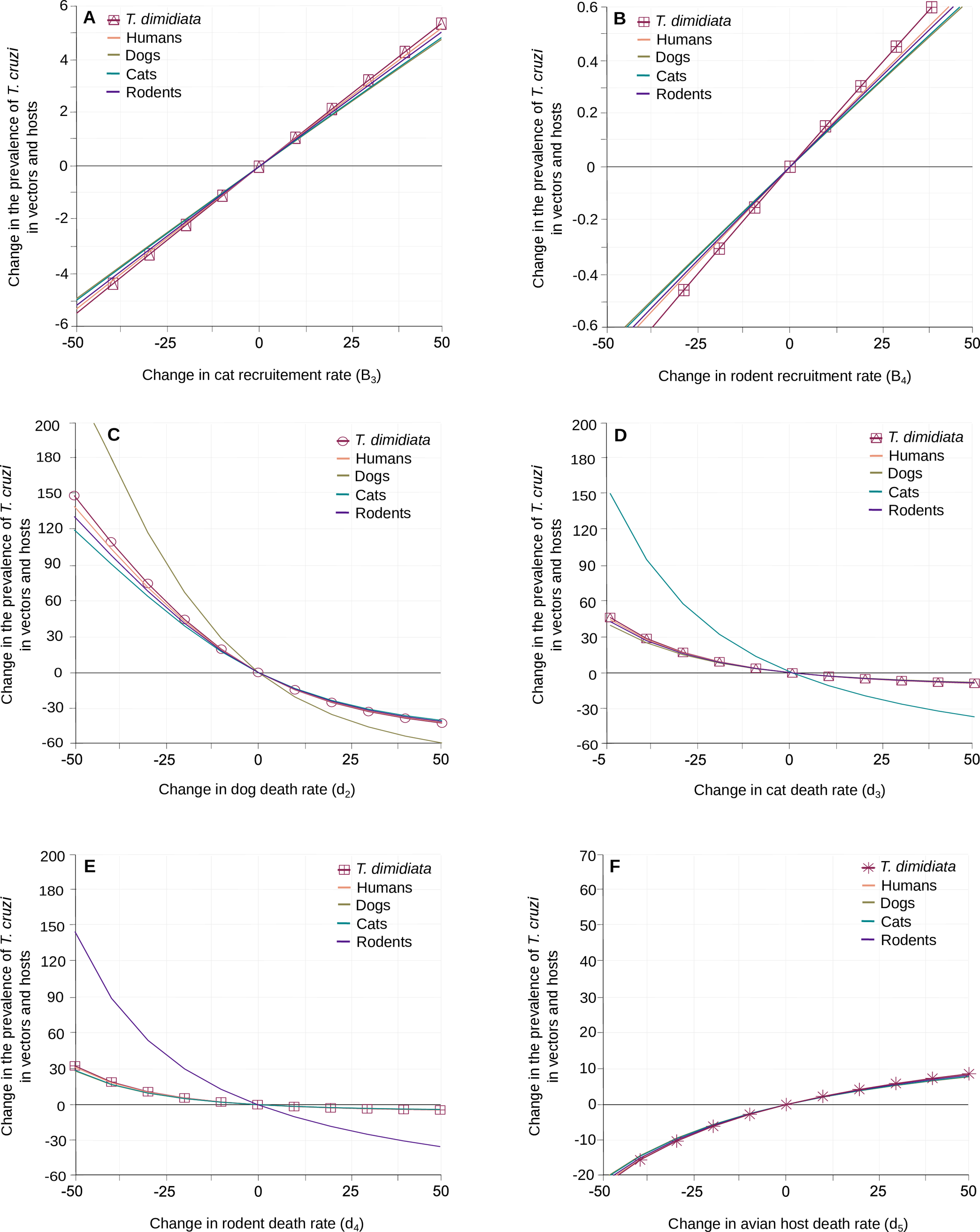

Supplement: S7 Appendix — (TIF) [file pntd.0007902.s007.tif]
